# *N*-Benzyl-*N*-(2-meth­oxy­phen­yl)benzene­sulfonamide

**DOI:** 10.1107/S1600536810043369

**Published:** 2010-10-30

**Authors:** Aziz-ur-Rehman, Wajeeha Tanveer, Mehmet Akkurt, Almas Sattar, Muhammad Athar Abbasi, Islam Ullah Khan

**Affiliations:** a Department of Chemistry, Government College University, Lahore 54000, Pakistan; b Department of Physics, Faculty of Arts and Sciences, Erciyes University, 38039 Kayseri, Turkey

## Abstract

In the title mol­ecule, C_20_H_19_NO_3_S, the dihedral angle between the phenyl rings is 48.93 (18)°, and they make dihedral angles of 38.37 (17) and 86.50 (19)° with the benzene ring. A weak intra­molecular C—H⋯O inter­action might stabilize the mol­ecular conformation. In the crystal, weak π–π stacking inter­actions between the benzene rings [centroid–centroid distance = 3.774 (2) Å] may help to establish the packing.

## Related literature

For background on the biological activity of sulfonamide derivatives, see: Ozbek *et al.* (2007 ▶); Parari *et al.* (2008 ▶). For the structures of some sulfonamide derivatives, see, for example: Asiri *et al.* (2009 ▶); Aziz-ur-Rehman *et al.* (2010 ▶).
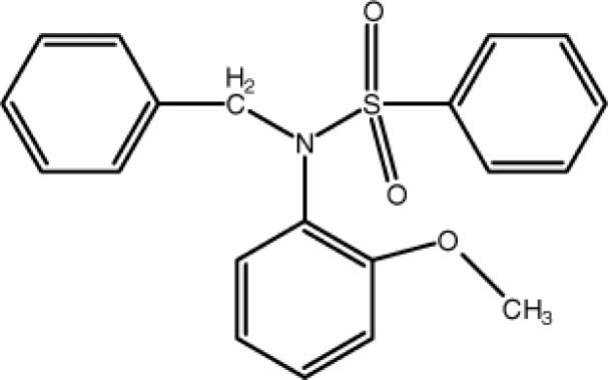


## Experimental

### 

#### Crystal data

C_20_H_19_NO_3_S
*M* *_r_* = 353.43
Monoclinic, 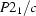

*a* = 10.0368 (3) Å
*b* = 9.0176 (3) Å
*c* = 20.4228 (7) Å
β = 103.424 (2)°
*V* = 1797.92 (10) Å^3^
*Z* = 4
Mo *K*α radiation
μ = 0.20 mm^−1^
*T* = 296 K
0.25 × 0.13 × 0.09 mm

#### Data collection

Bruker APEXII CCD diffractometer
16779 measured reflections
4438 independent reflections
2560 reflections with *I* > 2σ(*I*)
*R*_int_ = 0.037

#### Refinement

*R*[*F* ^2^ > 2σ(*F* ^2^)] = 0.061
*wR*(*F* ^2^) = 0.202
*S* = 1.04
4438 reflections
228 parameters
H-atom parameters constrained
Δρ_max_ = 0.35 e Å^−3^
Δρ_min_ = −0.20 e Å^−3^

### 

Data collection: *APEX2* (Bruker, 2007 ▶); cell refinement: *SAINT* (Bruker, 2007 ▶); data reduction: *SAINT*; program(s) used to solve structure: *SHELXS97* (Sheldrick, 2008 ▶); program(s) used to refine structure: *SHELXL97* (Sheldrick, 2008 ▶); molecular graphics: *ORTEP-3 for Windows* (Farrugia, 1997 ▶); software used to prepare material for publication: *WinGX* (Farrugia, 1999 ▶) and *PLATON* (Spek, 2009 ▶).

## Supplementary Material

Crystal structure: contains datablocks global, I. DOI: 10.1107/S1600536810043369/hb5703sup1.cif

Structure factors: contains datablocks I. DOI: 10.1107/S1600536810043369/hb5703Isup2.hkl

Additional supplementary materials:  crystallographic information; 3D view; checkCIF report

## Figures and Tables

**Table 1:** Hydrogen-bond geometry (Å, °)

*D*—H⋯*A*	*D*—H	H⋯*A*	*D*⋯*A*	*D*—H⋯*A*
C14—H14*B*⋯O3	0.97	2.36	2.972 (4)	120

**Fig. 1.**
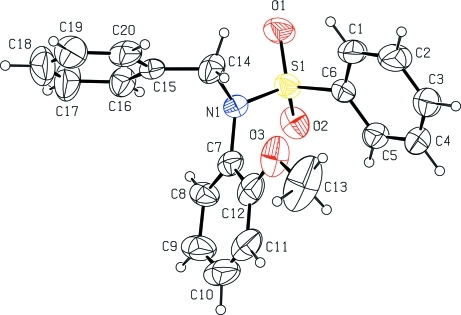
View of the title molecule with displacement ellipsoids drawn at the 30% probability level.

**Fig. 2.**
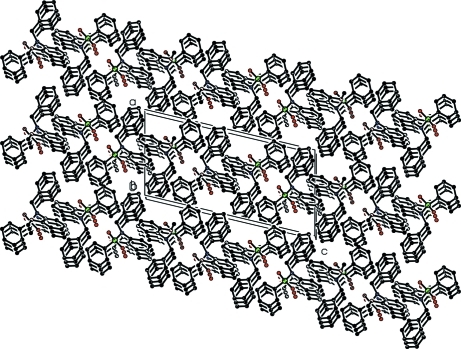
The packing of (I), viewing down the b axis in the unit cell. For clarity, all H atoms have been omitted.

## supplementary crystallographic information

### Comment

Sulfonamides are found in a number of natural as well as synthetic compounds. These molecules are considered as biologically very acitive compounds (Ozbek *et al.*, 2007, Parari *et al.*, 2008). As a contribution to a structural study of sulfonamide derivatives (Asiri *et al.*, 2009, Aziz-ur-Rehman *et al.*, 2010) here, we report the title compound (I).

In the molecule of (I), (Fig. 1), the bond lengths and bond angles are within the expected ranges. The geometry around S1 atom is distorted from a regular tetrahedron. The largest deviation is in the angle O2—S1—O1 [118.92 (12)°].

The dihedral angle between the phenyl rings (C1–C6) and (C15–C20) is 48.93 (18)° and they make dihedral angles of 38.37 (17) and 86.50 (19)°, respectively, with the benzene ring (C7–C12).

In the crystal structure, there is no classic hydrogen bonds. Weak intramolecular C—H···O interactions stabilize the molecular conformation. π–π stacking interactions [*Cg*2···*Cg*2 (1 - *x*, -*y*, 1 - *z*) = 3.774 (2) Å; *Cg*2 is a centroid of the C7–C12 benzene ring] contribute to the stabilization of the crystal structure. Fig. 2 show the packing diagram of (I) down the *b* axis.

### Experimental

A mixture of *N*-(2-methoxyphenyl)benzenesulfonamide (1.24 g, 5.0 mmol), sodium hydride (0.24 g, 10 mmol) and *N,N*-dimethylformamide (10 ml) was stirred at room temperature for 45 min and the benzyl chloride (0.64 g, 5.0 mmol) was added. The stirring was continued further for a period of 3 h and the contents were poured over crushed ice. The precipitated product was isolated, washed and re-crystallized from methanolic solution to yield light violet blocks of (I). Yield 65%.

### Refinement

All H atoms were positioned geometrically with C—H = 0.93–0.97 Å and allowed to ride on their parent atoms, with *U*_iso_(H) = 1.2 or 1.5*U*_eq_(C).

### Crystal data

**Table:**

C_20_H_19_NO_3_S	*F*(000) = 744
*M**_r_* = 353.43	*D*_x_ = 1.306 Mg m^−^^3^
Monoclinic, *P*2_1_/*c*	Mo *K*α radiation, λ = 0.71073 Å
Hall symbol: -P 2ybc	Cell parameters from 3872 reflections
*a* = 10.0368 (3) Å	θ = 2.5–21.8°
*b* = 9.0176 (3) Å	µ = 0.20 mm^−^^1^
*c* = 20.4228 (7) Å	*T* = 296 K
β = 103.424 (2)°	Block, light violet
*V* = 1797.92 (10) Å^3^	0.25 × 0.13 × 0.09 mm
*Z* = 4	

### Data collection

**Table:**

Bruker APEXII CCD diffractometer	2560 reflections with *I* > 2σ(*I*)
Radiation source: sealed tube	*R*_int_ = 0.037
graphite	θ_max_ = 28.4°, θ_min_ = 3.3°
φ and ω scans	*h* = −13→13
16779 measured reflections	*k* = −12→9
4438 independent reflections	*l* = −27→25

### Refinement

**Table:**

Refinement on *F*^2^	Primary atom site location: structure-invariant direct methods
Least-squares matrix: full	Secondary atom site location: difference Fourier map
*R*[*F*^2^ > 2σ(*F*^2^)] = 0.061	Hydrogen site location: inferred from neighbouring sites
*wR*(*F*^2^) = 0.202	H-atom parameters constrained
*S* = 1.04	*w* = 1/[σ^2^(*F*_o_^2^) + (0.1008*P*)^2^ + 0.2694*P*] where *P* = (*F*_o_^2^ + 2*F*_c_^2^)/3
4438 reflections	(Δ/σ)_max_ < 0.001
228 parameters	Δρ_max_ = 0.35 e Å^−^^3^
0 restraints	Δρ_min_ = −0.20 e Å^−^^3^

### Special details

**Table:**

Geometry. Bond distances, angles *etc*. have been calculated using the rounded fractional coordinates. All su's are estimated from the variances of the (full) variance-covariance matrix. The cell e.s.d.'s are taken into account in the estimation of distances, angles and torsion angles
Refinement. Refinement on *F*^2^ for ALL reflections except those flagged by the user for potential systematic errors. Weighted *R*-factors *wR* and all goodnesses of fit *S* are based on *F*^2^, conventional *R*-factors *R* are based on *F*, with *F* set to zero for negative *F*^2^. The observed criterion of *F*^2^ > σ(*F*^2^) is used only for calculating -*R*-factor-obs *etc*. and is not relevant to the choice of reflections for refinement. *R*-factors based on *F*^2^ are statistically about twice as large as those based on *F*, and *R*-factors based on ALL data will be even larger.

### Fractional atomic coordinates and isotropic or equivalent isotropic displacement parameters (Å^2^)

**Table:**

	*x*	*y*	*z*	*U*_iso_*/*U*_eq_	
S1	0.65180 (7)	0.44517 (7)	0.66852 (3)	0.0726 (2)	
O1	0.5815 (3)	0.5433 (2)	0.70356 (11)	0.1098 (9)	
O2	0.7233 (2)	0.5054 (2)	0.62194 (9)	0.0897 (7)	
O3	0.6440 (2)	0.0515 (2)	0.65783 (15)	0.1138 (9)	
N1	0.53841 (18)	0.3280 (2)	0.62738 (9)	0.0636 (6)	
C1	0.7345 (3)	0.2992 (4)	0.78758 (13)	0.0981 (13)	
C2	0.8243 (4)	0.2106 (5)	0.83247 (15)	0.1226 (18)	
C3	0.9455 (4)	0.1657 (4)	0.81816 (18)	0.1143 (14)	
C4	0.9776 (3)	0.2066 (4)	0.76011 (17)	0.0971 (11)	
C5	0.8896 (3)	0.2937 (3)	0.71487 (14)	0.0798 (10)	
C6	0.7687 (2)	0.3409 (3)	0.72877 (12)	0.0691 (8)	
C7	0.5817 (2)	0.2371 (3)	0.57779 (13)	0.0704 (9)	
C8	0.5683 (3)	0.2888 (3)	0.51265 (13)	0.0787 (10)	
C9	0.6088 (4)	0.2160 (5)	0.4631 (2)	0.1162 (17)	
C10	0.6693 (4)	0.0790 (7)	0.4816 (3)	0.146 (3)	
C11	0.6818 (3)	0.0179 (4)	0.5442 (3)	0.1157 (16)	
C12	0.6375 (3)	0.1012 (3)	0.5923 (2)	0.0933 (12)	
C13	0.7184 (5)	−0.0828 (4)	0.6767 (4)	0.197 (3)	
C14	0.4294 (3)	0.2778 (4)	0.65914 (14)	0.0898 (12)	
C15	0.2919 (2)	0.2704 (2)	0.61222 (11)	0.0596 (7)	
C16	0.2544 (3)	0.3576 (3)	0.55669 (14)	0.0846 (10)	
C17	0.1251 (4)	0.3467 (6)	0.51567 (19)	0.1411 (19)	
C18	0.0338 (4)	0.2513 (8)	0.5304 (2)	0.163 (3)	
C19	0.0679 (4)	0.1656 (6)	0.5857 (2)	0.1312 (18)	
C20	0.1961 (3)	0.1745 (3)	0.62612 (16)	0.0913 (12)	
H1	0.65250	0.33020	0.79690	0.1180*	
H2	0.80280	0.18120	0.87240	0.1470*	
H3	1.00590	0.10670	0.84880	0.1370*	
H4	1.05960	0.17550	0.75090	0.1170*	
H5	0.91140	0.32100	0.67470	0.0960*	
H8	0.52800	0.38130	0.50240	0.0940*	
H9	0.59720	0.25430	0.41990	0.1390*	
H10	0.70320	0.02610	0.44990	0.1750*	
H11	0.71880	−0.07620	0.55410	0.1390*	
H13A	0.66840	−0.16440	0.65250	0.2960*	
H13B	0.73040	−0.09860	0.72420	0.2960*	
H13C	0.80640	−0.07570	0.66590	0.2960*	
H14A	0.42480	0.34480	0.69570	0.1080*	
H14B	0.45290	0.18020	0.67840	0.1080*	
H16	0.31660	0.42500	0.54640	0.1020*	
H17	0.10080	0.40590	0.47750	0.1690*	
H18	−0.05330	0.24440	0.50230	0.1960*	
H19	0.00400	0.10060	0.59630	0.1570*	
H20	0.21930	0.11420	0.66400	0.1090*	

### Atomic displacement parameters (Å^2^)

**Table:**

	*U*^11^	*U*^22^	*U*^33^	*U*^12^	*U*^13^	*U*^23^
S1	0.0824 (4)	0.0642 (4)	0.0690 (4)	0.0044 (3)	0.0130 (3)	−0.0103 (3)
O1	0.1269 (17)	0.0989 (15)	0.0955 (13)	0.0447 (12)	0.0092 (12)	−0.0309 (12)
O2	0.1001 (13)	0.0788 (12)	0.0880 (12)	−0.0271 (10)	0.0174 (11)	0.0054 (10)
O3	0.0902 (15)	0.0710 (13)	0.164 (2)	−0.0034 (10)	−0.0035 (15)	0.0263 (14)
N1	0.0586 (10)	0.0718 (12)	0.0640 (11)	0.0007 (9)	0.0216 (8)	−0.0025 (9)
C1	0.097 (2)	0.134 (3)	0.0643 (15)	0.0410 (19)	0.0209 (14)	−0.0038 (17)
C2	0.138 (3)	0.166 (4)	0.0667 (18)	0.056 (3)	0.0298 (19)	0.014 (2)
C3	0.105 (2)	0.141 (3)	0.085 (2)	0.048 (2)	−0.0021 (18)	−0.011 (2)
C4	0.0692 (17)	0.115 (2)	0.103 (2)	0.0134 (16)	0.0119 (16)	−0.021 (2)
C5	0.0717 (16)	0.0852 (18)	0.0841 (17)	−0.0051 (14)	0.0212 (14)	−0.0140 (15)
C6	0.0692 (14)	0.0724 (15)	0.0631 (13)	0.0051 (11)	0.0098 (11)	−0.0146 (11)
C7	0.0557 (12)	0.0657 (15)	0.0943 (18)	−0.0151 (11)	0.0269 (12)	−0.0128 (13)
C8	0.0878 (17)	0.0890 (18)	0.0696 (15)	−0.0247 (14)	0.0391 (13)	−0.0114 (14)
C9	0.110 (3)	0.142 (3)	0.116 (3)	−0.047 (2)	0.066 (2)	−0.053 (3)
C10	0.094 (3)	0.162 (5)	0.203 (5)	−0.054 (3)	0.078 (3)	−0.108 (4)
C11	0.0668 (17)	0.076 (2)	0.206 (4)	−0.0154 (15)	0.035 (2)	−0.056 (3)
C12	0.0590 (14)	0.0666 (17)	0.152 (3)	−0.0187 (13)	0.0200 (17)	−0.018 (2)
C13	0.128 (3)	0.080 (3)	0.363 (9)	0.005 (2)	0.014 (4)	0.065 (4)
C14	0.0643 (14)	0.134 (3)	0.0764 (16)	0.0061 (15)	0.0273 (13)	0.0290 (17)
C15	0.0608 (12)	0.0625 (13)	0.0620 (12)	0.0023 (10)	0.0277 (10)	−0.0041 (10)
C16	0.0710 (15)	0.096 (2)	0.0869 (18)	−0.0005 (14)	0.0187 (13)	0.0136 (16)
C17	0.081 (2)	0.231 (5)	0.101 (2)	−0.001 (3)	0.0002 (19)	0.045 (3)
C18	0.074 (2)	0.298 (8)	0.110 (3)	−0.043 (3)	0.006 (2)	−0.013 (4)
C19	0.095 (2)	0.179 (4)	0.133 (3)	−0.061 (3)	0.054 (2)	−0.037 (3)
C20	0.094 (2)	0.096 (2)	0.100 (2)	−0.0161 (16)	0.0556 (17)	−0.0029 (17)

### Geometric parameters (Å, °)

**Table:**

S1—O1	1.424 (3)	C16—C17	1.375 (5)
S1—O2	1.426 (2)	C17—C18	1.341 (7)
S1—N1	1.6351 (19)	C18—C19	1.346 (7)
S1—C6	1.762 (2)	C19—C20	1.360 (5)
O3—C12	1.398 (5)	C1—H1	0.9300
O3—C13	1.428 (5)	C2—H2	0.9300
N1—C7	1.446 (3)	C3—H3	0.9300
N1—C14	1.467 (4)	C4—H4	0.9300
C1—C2	1.381 (5)	C5—H5	0.9300
C1—C6	1.376 (4)	C8—H8	0.9300
C2—C3	1.376 (6)	C9—H9	0.9300
C3—C4	1.350 (5)	C10—H10	0.9300
C4—C5	1.368 (4)	C11—H11	0.9300
C5—C6	1.376 (4)	C13—H13A	0.9600
C7—C8	1.386 (4)	C13—H13B	0.9600
C7—C12	1.351 (4)	C13—H13C	0.9600
C8—C9	1.346 (5)	C14—H14A	0.9700
C9—C10	1.390 (7)	C14—H14B	0.9700
C10—C11	1.371 (8)	C16—H16	0.9300
C11—C12	1.389 (6)	C17—H17	0.9300
C14—C15	1.488 (4)	C18—H18	0.9300
C15—C16	1.359 (3)	C19—H19	0.9300
C15—C20	1.371 (4)	C20—H20	0.9300
			
O1—S1—O2	118.92 (12)	C6—C1—H1	121.00
O1—S1—N1	107.03 (13)	C1—C2—H2	120.00
O1—S1—C6	107.94 (12)	C3—C2—H2	120.00
O2—S1—N1	107.55 (10)	C2—C3—H3	120.00
O2—S1—C6	108.22 (11)	C4—C3—H3	120.00
N1—S1—C6	106.57 (11)	C3—C4—H4	120.00
C12—O3—C13	115.8 (4)	C5—C4—H4	120.00
S1—N1—C7	116.09 (15)	C4—C5—H5	120.00
S1—N1—C14	118.28 (17)	C6—C5—H5	120.00
C7—N1—C14	120.7 (2)	C7—C8—H8	117.00
C2—C1—C6	118.8 (3)	C9—C8—H8	117.00
C1—C2—C3	120.1 (3)	C8—C9—H9	123.00
C2—C3—C4	120.6 (3)	C10—C9—H9	123.00
C3—C4—C5	120.1 (3)	C9—C10—H10	118.00
C4—C5—C6	120.0 (3)	C11—C10—H10	118.00
S1—C6—C1	119.66 (19)	C10—C11—H11	121.00
S1—C6—C5	119.81 (19)	C12—C11—H11	121.00
C1—C6—C5	120.4 (2)	O3—C13—H13A	109.00
N1—C7—C8	120.2 (2)	O3—C13—H13B	109.00
N1—C7—C12	122.5 (3)	O3—C13—H13C	109.00
C8—C7—C12	117.3 (3)	H13A—C13—H13B	110.00
C7—C8—C9	125.4 (3)	H13A—C13—H13C	109.00
C8—C9—C10	114.7 (4)	H13B—C13—H13C	110.00
C9—C10—C11	123.4 (5)	N1—C14—H14A	109.00
C10—C11—C12	117.9 (4)	N1—C14—H14B	109.00
O3—C12—C7	115.2 (3)	C15—C14—H14A	109.00
O3—C12—C11	123.5 (3)	C15—C14—H14B	109.00
C7—C12—C11	121.3 (4)	H14A—C14—H14B	108.00
N1—C14—C15	113.9 (2)	C15—C16—H16	120.00
C14—C15—C16	123.2 (2)	C17—C16—H16	120.00
C14—C15—C20	119.0 (2)	C16—C17—H17	120.00
C16—C15—C20	117.8 (2)	C18—C17—H17	120.00
C15—C16—C17	120.4 (3)	C17—C18—H18	120.00
C16—C17—C18	120.4 (4)	C19—C18—H18	120.00
C17—C18—C19	120.2 (4)	C18—C19—H19	120.00
C18—C19—C20	119.7 (4)	C20—C19—H19	120.00
C15—C20—C19	121.4 (3)	C15—C20—H20	119.00
C2—C1—H1	121.00	C19—C20—H20	119.00
			
O1—S1—N1—C7	168.04 (17)	C3—C4—C5—C6	0.6 (5)
O2—S1—N1—C7	39.19 (19)	C4—C5—C6—S1	−176.8 (2)
C6—S1—N1—C7	−76.67 (18)	C4—C5—C6—C1	−1.0 (4)
O1—S1—N1—C14	−36.5 (2)	C12—C7—C8—C9	−1.4 (5)
O2—S1—N1—C14	−165.4 (2)	N1—C7—C12—O3	3.1 (4)
C6—S1—N1—C14	78.8 (2)	N1—C7—C12—C11	−178.2 (3)
N1—S1—C6—C1	−79.3 (3)	N1—C7—C8—C9	177.9 (3)
O1—S1—C6—C1	35.3 (3)	C8—C7—C12—O3	−177.6 (2)
O2—S1—C6—C1	165.3 (2)	C8—C7—C12—C11	1.1 (4)
N1—S1—C6—C5	96.5 (2)	C7—C8—C9—C10	−0.6 (6)
O1—S1—C6—C5	−148.8 (2)	C8—C9—C10—C11	3.0 (7)
O2—S1—C6—C5	−18.9 (3)	C9—C10—C11—C12	−3.3 (7)
C13—O3—C12—C11	9.1 (5)	C10—C11—C12—O3	179.7 (3)
C13—O3—C12—C7	−172.2 (3)	C10—C11—C12—C7	1.1 (5)
C7—N1—C14—C15	−67.7 (3)	N1—C14—C15—C16	−27.6 (4)
C14—N1—C7—C12	−62.3 (3)	N1—C14—C15—C20	154.3 (2)
S1—N1—C7—C12	92.5 (3)	C14—C15—C16—C17	−179.2 (3)
C14—N1—C7—C8	118.5 (3)	C20—C15—C16—C17	−1.2 (4)
S1—N1—C14—C15	138.1 (2)	C14—C15—C20—C19	178.6 (3)
S1—N1—C7—C8	−86.7 (2)	C16—C15—C20—C19	0.4 (4)
C2—C1—C6—S1	176.5 (3)	C15—C16—C17—C18	0.9 (6)
C2—C1—C6—C5	0.6 (5)	C16—C17—C18—C19	0.2 (8)
C6—C1—C2—C3	0.1 (6)	C17—C18—C19—C20	−1.0 (8)
C1—C2—C3—C4	−0.5 (6)	C18—C19—C20—C15	0.7 (6)
C2—C3—C4—C5	0.1 (5)		

### Hydrogen-bond geometry (Å, °)

**Table:**

*D*—H···*A*	*D*—H	H···*A*	*D*···*A*	*D*—H···*A*
C14—H14B···O3	0.97	2.36	2.972 (4)	120
